# A Likelihood-Based Approach to Identifying Contaminated Food Products Using Sales Data: Performance and Challenges

**DOI:** 10.1371/journal.pcbi.1003692

**Published:** 2014-07-03

**Authors:** James Kaufman, Justin Lessler, April Harry, Stefan Edlund, Kun Hu, Judith Douglas, Christian Thoens, Bernd Appel, Annemarie Käsbohrer, Matthias Filter

**Affiliations:** 1 IBM Almaden Research Center, San Jose, California, United States of America; 2 Department of Epidemiology, Johns Hopkins Bloomberg School of Public Health, Baltimore, Maryland, United States of America; 3 Federal Institute for Risk Assessment, Berlin, Germany; Pennsylvania State University, United States of America

## Abstract

Foodborne disease outbreaks of recent years demonstrate that due to increasingly interconnected supply chains these type of crisis situations have the potential to affect thousands of people, leading to significant healthcare costs, loss of revenue for food companies, and—in the worst cases—death. When a disease outbreak is detected, identifying the contaminated food quickly is vital to minimize suffering and limit economic losses. Here we present a likelihood-based approach that has the potential to accelerate the time needed to identify possibly contaminated food products, which is based on exploitation of food products sales data and the distribution of foodborne illness case reports. Using a real world food sales data set and artificially generated outbreak scenarios, we show that this method performs very well for contamination scenarios originating from a single “guilty” food product. As it is neither always possible nor necessary to identify the *single* offending product, the method has been extended such that it can be used as a binary classifier. With this extension it is possible to generate a set of potentially “guilty” products that contains the real outbreak source with very high accuracy. Furthermore we explore the patterns of food distributions that lead to “hard-to-identify” foods, the possibility of identifying these food groups *a priori*, and the extent to which the likelihood-based method can be used to quantify uncertainty. We find that high spatial correlation of sales data between products may be a useful indicator for “hard-to-identify” products.

## Author Summary

Response to foodborne disease outbreaks is complicated by globalization of our food supply chains. Rapid identification of contaminated products is essential to limit the damage caused by foodborne disease. Worldwide, foodborne disease outbreaks are responsible for $9B a year in medical costs and over $75B in economic losses. Yet relevant data required to accelerate the identification of suspicious food already exists as part of the inventory control systems used by retailers and distributors today. Combining this retail data with public health case reports has the potential to hasten outbreak investigations and provide public health investigators with better information on suspected products to test. This paper demonstrates the feasibility of the principle and efficiency of this approach. Based on these findings it can be concluded that in foodborne disease outbreaks retail data could be used to speed and target public health investigations and consequently reduce numbers of sick/dead people as well as reduce economic losses to the industry.

## Introduction

In recent years global trade has significantly altered the topology of food supply chains [1]. As a result, the potential impact of contamination events has increased [2]. Worldwide, foodborne illness causes billions of dollars in healthcare related costs each year [3], and more in economic losses to farmers, distributors, and food retailers [4], [5]. In case of a foodborne disease outbreak, rapid identification of contaminated products is essential, since the medical and economic damages incurred grow with the duration of the outbreak. Currently public health investigators must reconstruct the relevant food distribution network in order to identify the contaminated food product or contaminated product groups during an outbreak [6]. Lab-based analytical methods frequently provide the “gold standard” in verifying the source of foodborne illness outbreaks. These methods verify or cast doubt on epidemiological findings originating from case control studies with food consumption questionnaires [7]. In addition, the ability to track food through different stages of production, processing, and distribution (traceability) has been the subject of extensive study [8], [9]. Nevertheless the time required to accomplish such investigations usually ranges from weeks to months. Accelerating this process may reduce the number of people sickened and help to restore consumer confidence in the safety of food products [10].

In a previous study, as a possible strategy to achieve this goal, we proposed a likelihood-based method that could be applied as an early response system to help determine the product most likely to be associated with a foodborne disease outbreak [11]. The method was tested with synthetic food sales data, but real data is readily available from retail sales companies. Proactive analysis of this retail data could complement and guide laboratory testing and trace back analysis.

In the work reported here, we test our likelihood-based method using raw food sales data. As a simplifying assumption, we model food consumption at the point of sale region. In future work, we will test this assumption by applying Huff's “gravity model” for retail shopping to smooth the sales distribution over other regions [12]. Smoothing the sales distribution will also allow sensitivity analysis to spatial noise in the case reports.

In applying the likelihood-based method to real world sales data, we use a ROC (receiver operating characteristics) analysis to quantify the performance of the method, comparing two different classifiers. This analysis also identifies the optimal discrimination threshold to maximize performance as a function of both the selectivity and specificity for the likelihood-based analysis. Additionally we explore how the method's performance may depend on “structural” properties of the sales data distribution, as this understanding is essential for efforts to proactively predict which contaminated foods/food groups might be hard to pinpoint in the event of an outbreak.

## Methods

### Food Sales Data

We apply product specific retail sales data from stores of a German food retail company covering 3,513 of Germany's 8,235 postal zones. The dataset lists the weekly sales of 580 anonymous food products (N = 580). For application in this analysis, sales data were aggregated per postal zone and product over the three-year period 01/2008 to 12/2010. Let *sales(n, r)* represent the number of units of food product *n* sold in region *r* over this three-year period. We can now define a function *f_s_(n, r)* representing the probability that product *n* is sold in region *r* as:
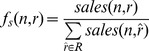
(1)where *R* is the set of all regions included in the analysis.

### Outbreak Pattern Generation

The underlying assumption of outbreak pattern generation is that for each product the distribution of sales across the postal codes reflects the true consumption pattern for that food [12]. Hence, the function *f_c_(n, r)* represents the probability that product *n* is *consumed* in region *r* and in this paper we simply assume probability of consumption equals probability of sale:
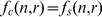
(2)


Notice that for a given product *n*, *f_c_ (n, r)* is a discrete probability mass function representing the probability that product *n* is consumed in location *r*, and that:
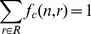
(3)


**Figure 1:**
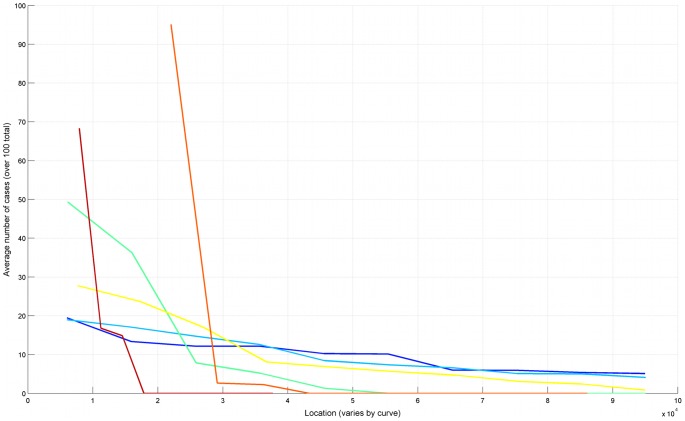
Number of case reports per location for several different outbreaks each generated based on a different product. For each product the results are averaged over 50 trials. For each trial, the x axis is sorted from most to least frequently occurring location to show the outbreak pattern.

We take advantage of this when generating synthetic outbreak case reports for a selected “contaminated” product *x* (where we use *x* instead of *n* to indicated a single contaminated product). Using A. J. Walker's alias method [13], we draw *M* random locations by sampling from *f_c_ (x, r)* over all locations *r* in *R*. In separate trials, synthetic case report data are generated assuming each of the 580 products, in turn, as the source of contamination. We assume the products are independent so *f_c_ (x, r)* also defines the probability of a case report at location *r* due to contaminated product *x*. It is true that two “products” with different local “brands” or “ids” could in fact be the same food item simply rebranded when repackaged locally. Conversely, a product sold on a national scale under one single brand could become contaminated at a single point of sale retail site (e.g., a butcher shop). For the purposes of this study, the simulated case reports were generated self consistently from the retail data using the assumption that the data provided to us by product id were independent. Depending upon the spatial distribution of product *x*, it is likely that, during one simulated outbreak of 100 cases, multiple case reports will come from a same postal code. Figure 1 plots the number of case reports per location for several different outbreaks each generated based on a different product. Distributions generated from widely distributed products (shown in blue) are flatter than distributions generated from products sold only locally or regionally (shown in red).

### Identifying Implicated Products

An outbreak can be described by the set of locations {*R*} of all reported cases where *r_i_* is the location of the *i^th^* case. Note that there is no limit or constraint on how many cases may come from a particular location. In order to identify implicated products we describe two estimation methods below.

#### Method 1: The likelihood based method

Let 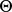 be a parameter vector of length N, such that 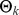 is 1 if a product *k* is contaminated and zero otherwise. Here we assume there is a single contaminated product in a given outbreak so only one element of vector 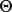. If we consider 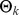 to be the parameter vector designating *k* as the contaminated product, then the likelihood of 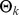 after observing *m* case reports is:
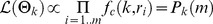
(4)where 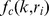 is the probability that an individual living in location *r_i_* consumed product *k* (see Text S1 on the derivation of likelihood). Hence each element *P_k_(m)* of the vector *P(m)* is proportional to the likelihood that product *k* is the contaminated product. Dividing each element of *P(m)* by the largest element in *P(m)* yields the likelihood ratio for each product being the contaminated product given the first m elements of *R*. We denote this as 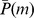. The product *k* that corresponds to the maximal element of 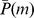 is our maximum likelihood estimate for the contaminated product.

#### Method 2: The pair-wise Spearman's rank correlation method

Let *sales(k)* be a vector of length 3,513 (number of postal zones or locations used) where each element represents the total number of units of product *k* sold in a given location. Also, assume *outbreak(R)* is also a vector of length 3,513 where each element represents the number of times a location was drawn in *R*. Now a pair-wise Spearman's rank correlation coefficient was calculated for element *k* in positive definite vector *P(m)* by:
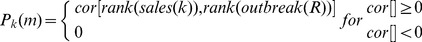
(5)


In this method, normalization of *P(m)* is done by setting its *k_th_* element to:
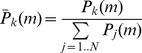
(6)


### Performance Estimation

We run the analysis varying the contaminated product, *x*, over all *N* = 580 products, and up to *M* = 100 synthetic case reports ending up with 58,000 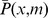 vectors. Next, we repeat the experiment over *S* = 100 randomly seeded runs, denoting 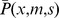 the outcome of 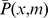 in the *s_th_* experiment. (See Dataset S1 and Dataset S2.) Now we define a statistic *A_x,m_* as such:
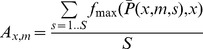
(7)

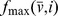
 is a function that returns 1 if the index of maximum element in vector *v* is *i*; if not it returns 0. We call statistic *A* the success rate [11].

Statistic *B* is based upon an ROC analysis. In an ROC analysis, we compute the average true positive rate and false positive rate (also called sensitivity and specificity). The average true positive rate (TPR) for a discrimination threshold ***t*** is defined as:
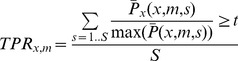
(8)


Here we assume the ≥ test returns 1 when satisfied, 0 otherwise. Essentially we sum the total number of outcomes where the ratio of “guilty” product *x* is above the threshold and then average over the *S* runs.

To define the false positive rate for a contaminated product *x* after *m* case reports, we first compute the number of true negatives in run *s*:
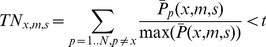
(9)


Next we compute the number of false positives:
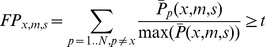
(10)


The average false positive rate is now defined as:
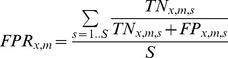
(11)


In the analysis, we use the thresholds *t* of 1/256, 1/128, 1/64, 1/32, 1/16, 1/8, 1/4, 1/2 and 1 to generate the Area Under Curve (AUC) statistic. As some food distributions within the data set had no overlap with the generated outbreak pattern, and to avoid overestimation of specificity, we exclude so-called “zero probability” products from the average in the corresponding scenario. A product belonged to the zero probability category, by definition, when after 100 trials and 100 case reports for each trail, that product is *never* sold in any sampled location. Failure to exclude the zero probability set would artificially exaggerate the specificity of the method.

### Clustering of Food Products

In order to analyze how different food distribution patterns can influence the performance of the likelihood-based method, the similarity of the distribution patterns of the food products was measured by calculating the pair-wise Spearman's rank correlation coefficient, 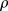, on the basis of sales distribution data of all food products [14]. Similar to the estimation technique described in method 2 above, let *sales(k)* be a vector of length 3,513 (number of locations) where each element is the total sales of product *k* in a given location. The pair-wise Spearman's rank correlation is between two products, *k* and *l* becomes:
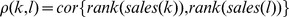
(12)


Since Spearman's 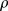 provides a measure of pair-wised association between food distributions, the value of 1−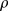 served as a *dissimilarity* measure describing the “distance” between each pair of food products. This measure was used as input for a hierarchical clustering algorithm [function hclust()] using the complete linkage method provided by the R-Manual [15].

## Results/Discussion

### Performance of the Likelihood-Based Method

In order to evaluate its performance the method has been applied to a real world dataset of 580 food products with known distribution patterns across Germany [11]. In this analysis the simplifying assumption has been made, that exactly one of the known food products is responsible for a disease outbreak, which were generated based on the corresponding “guilty” food product distribution. The number of sampled cases defining the outbreak size has been varied from 1 to 100.

**Figure 2:**
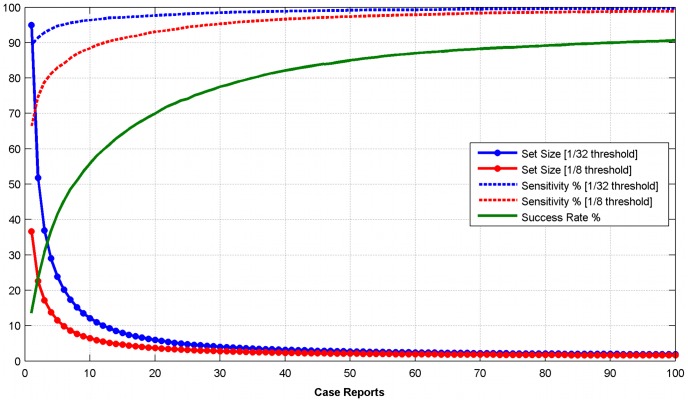
Average success rate, sensitivity with 1/32 and 1/8 threshold, and average suspect product set size vs. number of case reports.

To assess the performance of the likelihood-based method statistic *A* and *B* were used. Each statistic describes the capability of the method to correctly identify the source of infection back from the comparison of the artificial outbreak pattern with each of the 580 food products under investigation. In Figure 2 the green curve shows the success rate (statistic *A*) averaged over the 580 food products as contamination source. The average success rate of the algorithm rises steeply with the number of case reports reaching a level above 80% with only 50 case reports. However there are outbreak patterns for which the likelihood method is not effective with many more case reports required for unique identification of the correct “guilty” product. This is in line with the expectation the highest likelihood criteria are hard to accomplish for similarly distributed products.

Taking advantage of the likelihood-based approach we can also assess the relative probability for *all products*. Selecting a *discrimination threshold*, we can then identify the group or subset of all products with likelihood ratio greater than that threshold, which we call the “suspect product set”. In Figure 2 we also show the average probability that the contaminated product is found within this set for thresholds of 1/8 (cyan) and 1/32 (red). Also shown in Figure 2 is the number of products found (on average) within the suspect product set, as a function of the number of case reports, for the same choices of threshold. Even for a threshold of 1/32, the average set size falls to as few as a dozen suspect products within only ten case reports [16].

**Figure 3a–c:**
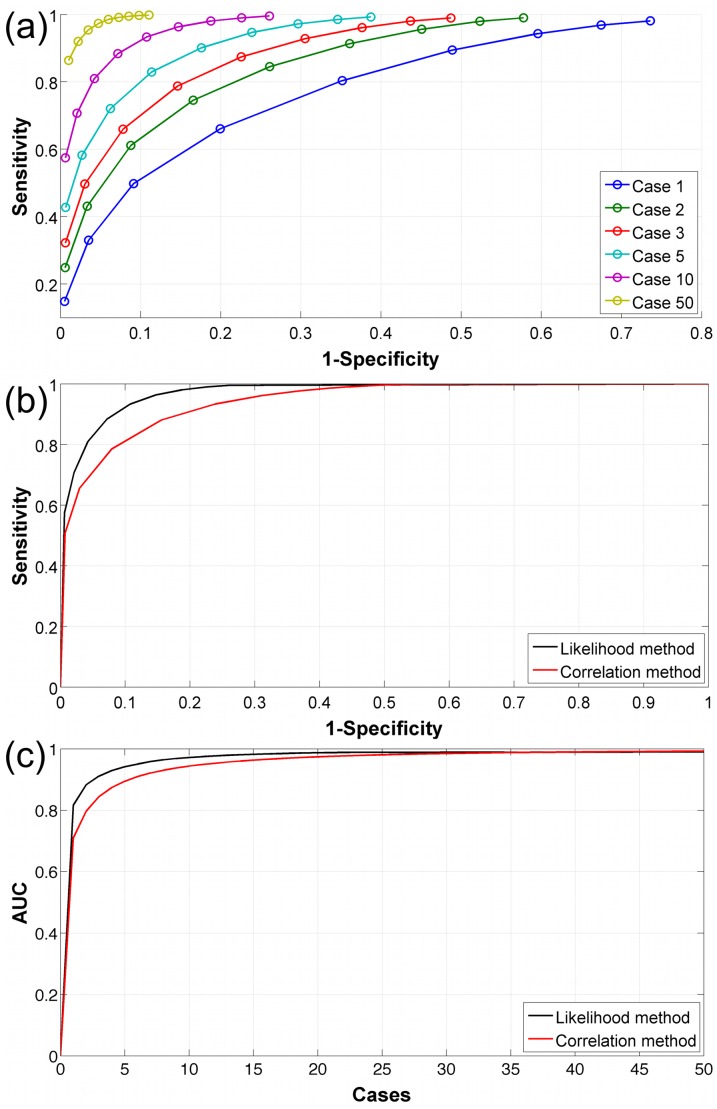
Performance measures of the likelihood-based approach. Figure 3a shows the ROC curve as a function of the number of case reports (see legend). Figure 3b shows a comparison of ROC curves generated with the likelihood-based method vs. a Spearman rank correlation based measure for outbreak patterns with 10 case reports. Figure 3c provides the area under the curve (AUC) as a function of the number of case reports for both classifiers.

To visualize the performance statistic *B* of this likelihood-based approach, we plot in Figure 3a the “receiver operating characteristic” or ROC curves for outbreak patterns with different numbers of cases. The ROC analysis characterizes the performance of the algorithm when the calculated likelihood ratio is applied as a binary classifier. The curve shows the “sensitivity” of the classifier as a measure of the fraction of true positives vs. the fraction of false positives (1-specificity). An ideal or perfect classifier would have a sensitivity of 1.0 at (1-specifity) = 0 (no false positives). The area under the ROC curve (AUC) provides a measure of overall performance. A perfect classifier has an AUC = 1.0. A useless classifier (e.g., with a linear ROC curve and slope of ½) would have an AUC of 0.5. As expected, this type of performance measure illustrates that the results of the likelihood-based approach depend on the number of case reports. Thus separate curves are shown for outbreaks with 1, 2, 3, 5, 10, and 50 cases. (The ROC curve is defined for only one case report. However, from a public health perspective an “outbreak” of foodborne illness is declared only after two or more cases.) As Figure 3a shows, the area under the cure approaches 1 for outbreak patterns with as few as 50 case reports. In Figures 3b and 3c, we compare the performance of the likelihood-based approach with a simple classifier based on the Spearman rank correlation coefficient 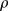. As these three figures make evident, the likelihood-based method outperforms the correlation-based approach. In a real world application, these performance improvements are of utmost importance to avoid false accusations of food manufacturers, unjustified product recalls, and a waste of limited analytical resources.

**Figure 4a–c:**
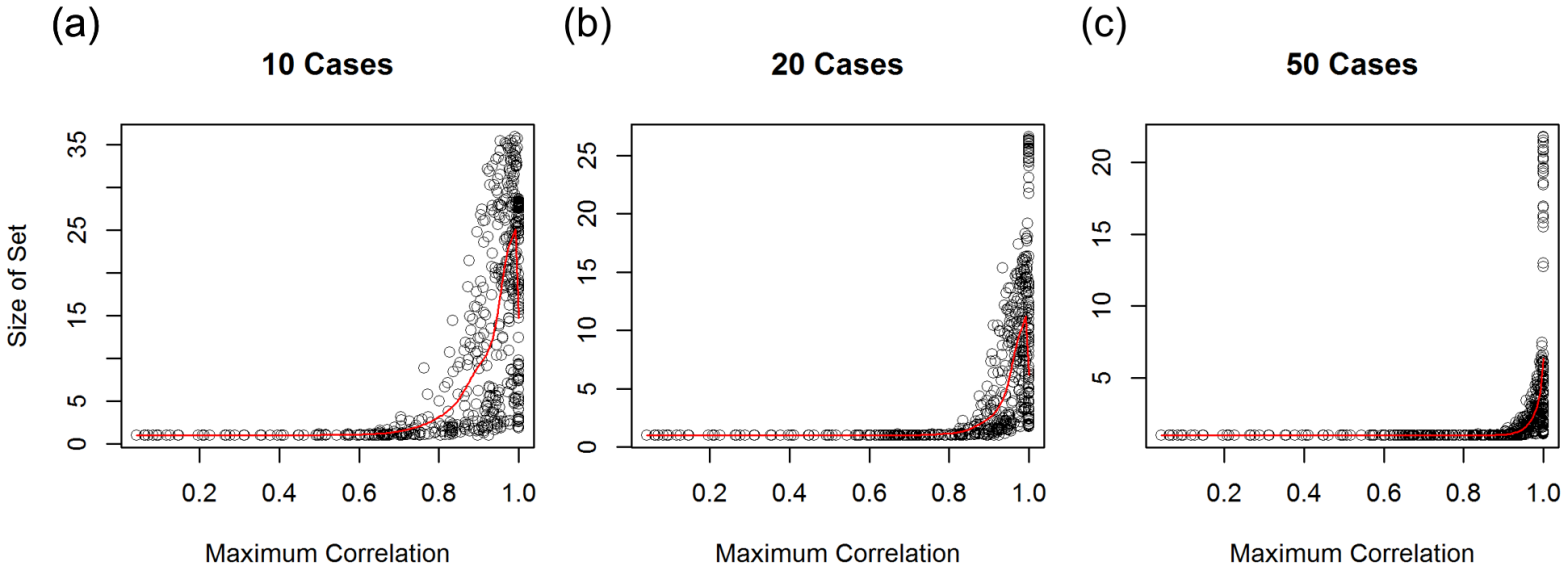
Suspect product set size vs. maximum pair-wise product correlation after observing 10, 20, and 50 simulated cases. For large correlation, the contaminated product cannot always be uniquely determined.

Using the Spearman's rank correlation coefficient 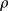, we explore how the performance of the likelihood-based method is related to associations between distinct product sales distributions. As Figure 4 and Figures S1 and S2 confirm, the algorithm's performance is strongly influenced by associations between food sales distributions. Plotting the maximum Spearman's 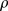 for each product against success rate, we assess how the magnitude of the association between the contaminated product and the food to which it is most similarly distributed affects the suspect product set size determined by the likelihood-based approach.

The data in Figure 4 demonstrates that the number of suspect products increases steeply if the contaminated food and the product most related to it have high correlation. The knee of the curve shifts with set size increasing sharply for correlation ≳ 0.8 given 10 case reports, ≳ 0.9 given 20 case reports, etc. Comparing Figure 4c to 4a, it is clear that as the maximum pairwise correlation between a contaminated product and another product increases, the number of cases required to *reduce* the suspect product set size to a manageable number (e.g., below 10) increases. In Figure S2 we also show the corresponding decrease in the “success rate” measure.

Consider ‘Y’ products with *identical* sales distributions. When the rank ordered distribution patterns of the contaminated food and at least one of these foods are equal, then the value of the likelihood for those products will remain the same. In this limit, the size of the suspect product set will never fall below Y, independent of the number of case reports. Understanding the maximum number of highly correlated products is therefore important given the larger goal of accelerating foodborne disease investigations, as it forewarns public health investigators of the largest number of products that may have to be tested together (in a worst case scenario).

### Clustering

**Figure 5:**
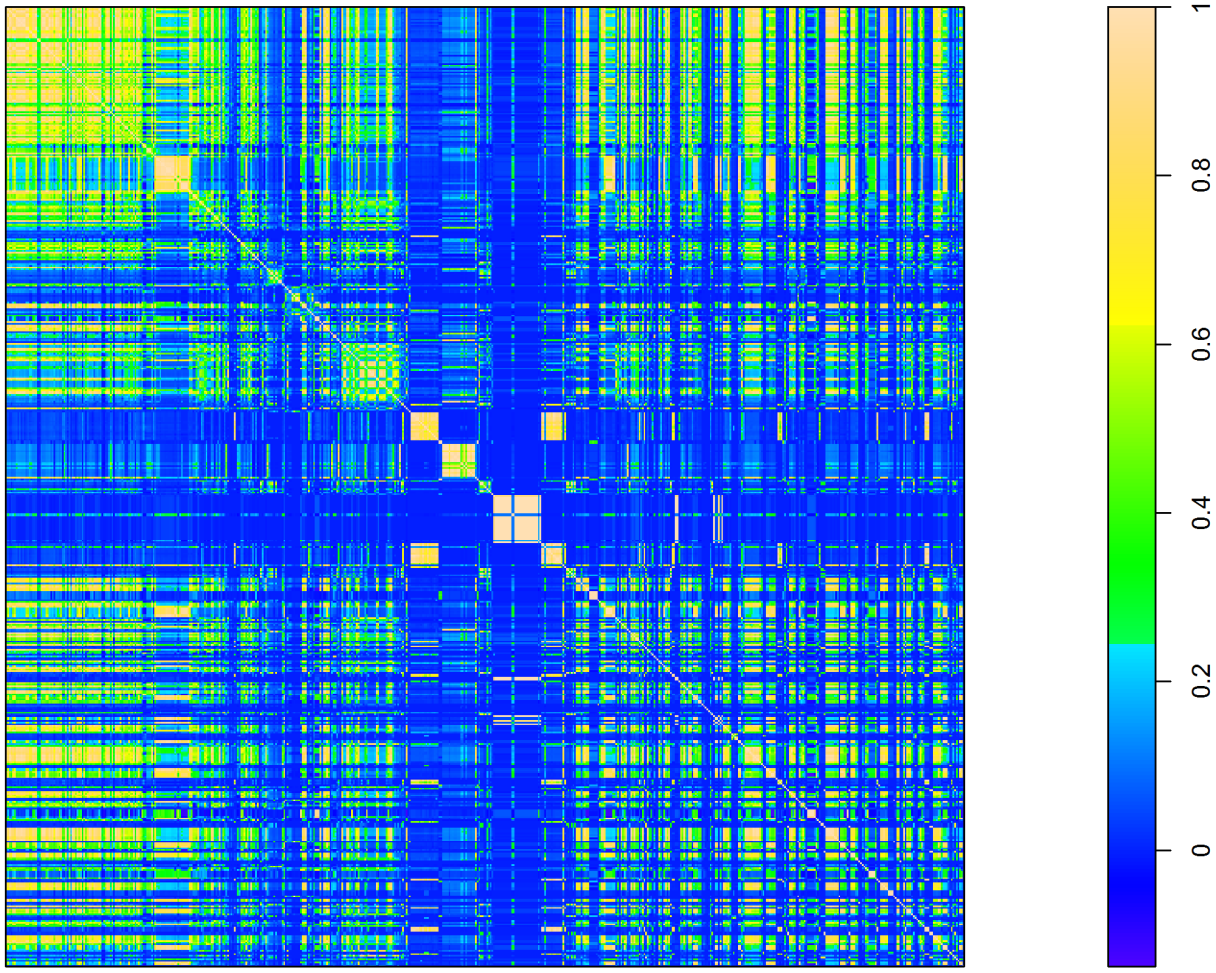
Heat map of the pair-wise Spearman's 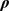 matrix. This figure depicts the correlation matrix map sorted by clusters.

**Figure 6:**
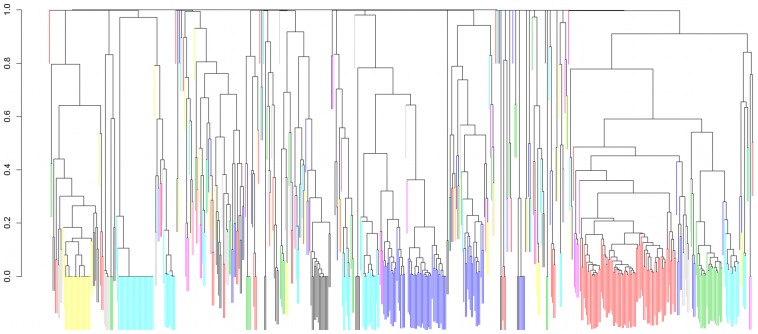
Hierarchical clustering diagram of 580 food products. Different colors indicate different clusters, defined by a cut-off value of 0.25. (Note that colors were used multiple times, i.e., non-adjacent clusters of the same color are not related in any special way.)

As noted, a high degree of similarity between the distribution patterns of the food products under investigation and the spatial pattern of the contaminated “guilty” product implies that it is (will be) difficult to correctly identify the causative food item. To describe and visualize this property of the food data set, we calculate the correlation matrix and apply hierarchical clustering algorithms. Figure 5 is a graphical representation of the pair-wise Spearman's correlation coefficient matrix as a so-called heat map. In this representation, products were sorted by the hierarchical clustering indicated in Figure 6. The colors indicate the degree of similarity between food products as measured by the Spearman's 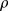. This representation supports the finding that there is a large cluster of highly similar distributed food products within the given data set. Products belonging to this cluster make the biggest contribution to rapid decrease in classifier performance when the number of case reports falls below 10 (data not shown). The figure shows a distribution of cluster sizes within the retail sales data.

Figure 6 shows a dendrogram visualizing the dissimilarity of the spatial distribution patterns of the 580 food products under investigation. Most similarly distributed food products are grouped at the bottom of the tree with a dissimilarity score close to 0 (i.e., the spatial distribution pattern is almost identical). Clusters of similar distributed food products are connected according to the dissimilarity score generated by the complete linkage method. For further investigations distinct clusters were generated (indicated by different colors) by cutting the tree horizontally at the dissimilarity level of 0.25. This ensures that within each cluster, all pair-wise Spearmen's correlation coefficients 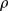 are at least 0.75. This choice of threshold was inspired by the observation reported above, which the suspect product set size increases rapidly when the maximum Spearman's 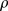 is above ∼0.8.

The *product* data used in this study was provided as point of sale retail data by anonymized product id. After completion of the study, the products were identified as various dairy products. The 580 food items include some items that are locally branded (and sold) and some very widely distributed products sold nationally. The only factor we could identify as important to the product clustering shown in Figure 6 was the spatial pattern of the food distribution including whether the food item was sold locally, regionally, or nationally. Categories, such as fresh or frozen, do not affect the observed clustering (and those factors where not used in generating the simulated outbreaks as they were not known to the authors before the study and not built into the simulation).

**Figure 7:**
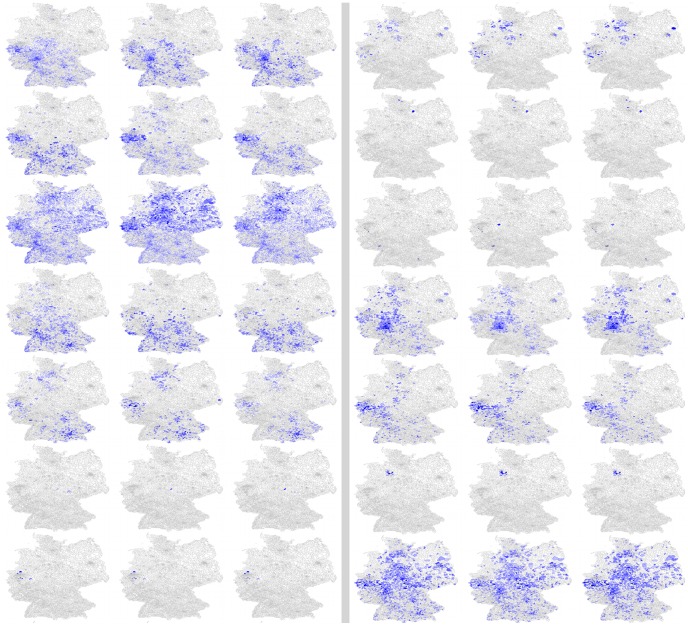
A series of small images illustrating distribution patterns of food products sold by a German food retail company stratified by zip codes. For illustration purposes, all product clusters containing exactly three products are displayed. Clusters are arranged in two columns of seven clusters each. Other cluster sizes exhibit similar correlations between product distribution patterns. This image is published with permission from Esri and its data providers, and from Michael Bauer Research GmbH, Nürnberg, Germany; Data Source: Microm 2013.

To characterize the clusters observed in the dendrogram in Figure 6, we show in Figure 7, for all clusters containing three food products, a series of small images showing color coded product sales volume in each of the 3,513 postal code regions where food is sold by the food retail company. The images are organized according to the product grouping generated by the clustering algorithm. The figure clearly shows that product clustering strongly depends on how widely spread or how localized is the spatial sale pattern of the product for each cluster. Products with similar sales distributions are placed in common clusters by the pair-wise Spearman's rank correlation method.

### Performance of the Likelihood-Based Method in Case of Food Product Clusters

**Figure 8a–c:**
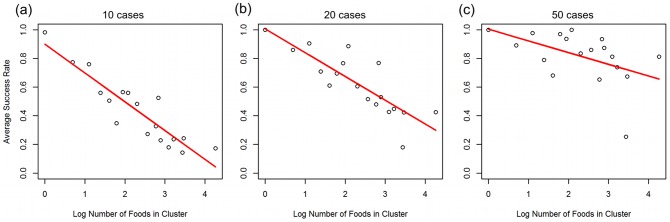
Average success rate after observing 10(a), 20(b), and 50(c) simulated cases vs. Log Number of foods in each cluster.

Figures 8 a–c show that the average success rate for identification of contaminated foods within a cluster of a certain size is linearly related to the log of cluster size for (a) 10 case reports, (b) 20 case reports, and (c) 50 case reports. It can be stated, that the *absolute magnitude* of the slope of this linear relationship *decreases* in the presence of larger numbers of cases. This confirms, that even for highly correlated food distribution patterns the performance of a likelihood-based classifier will increase with additional information on case reports.

### Conclusions

This analysis shows how, when information on the food distribution channels is available, likelihood-based methods can quickly identify those products likely to be causing an outbreak using the geographic locations for even relatively few cases. However, these methods assume that food distribution channels are well characterized, which may rarely be the case. Nevertheless, our methods could be extremely useful for retail companies that want to assess which of their own products could potentially be involved in an ongoing disease outbreak, or identifying chains or individual stores that should be prioritized for investigation in an ongoing outbreak. In practice, multiple products may be contaminated by a single food ingredient. Here we use a very simple model of the probability of individuals consuming food for particular shops, which may be quite different from real consumption patterns.

In this paper we also make the simplifying assumption that food is consumed where it is sold. In fact, people travel. In the future, it is possible to extend the current work by adding Huff's “gravity model” for retail shopping behavior [12]. This will effectively smooth the sales distribution over nearby regions. It will also make it possible to test the addition of noise in the case report generator. In the simplified model, any case report occurring in region where a product is never sold (probability 0) immediately excludes that product from consideration. The performance of the likelihood-based method in these more challenging scenarios will be explored in future research.

This analysis also provided some fundamental insights into the relationship of method's performance and inherited properties of the analyzed food sales data. We could confirm that the degree in similarity of the spatial food distribution pattern determines how quickly the likelihood method will converge on a finite suspect product set size. Generally, the maximum pair-wise correlation with the actual contaminated product is negatively related to success rate, and positively related to the number of cases required for a perfect prediction. This suggests that it may be beneficial to consider identifying groups of products as likely to contain the tainted food, rather than focusing on finding one product.

Additionally it has been shown that relevant intrinsic properties of the food sales data can be visualized by performing hierarchical clustering algorithms. This method provides a helpful graphical summary of the spatial similarity of food distributions. Further, on the basis of clusters generated by this algorithm, it is shown that log cluster size has a negative, linear relationship with success rate. This suggests that, as the number of products similarly distributed as the contaminated product increases, our ability to consistently identify the contaminated food in a small number of cases decreases. Highly correlated food product distributions are associated with products that are (and will be) harder to identify than uncorrelated product distributions. Since correlated product clusters can be identified proactively, suspect products can also be grouped for analysis accelerating an outbreak investigation.

## Supporting Information

Dataset S1 **This csv file contains normalized sales data for 580 anonymous food products across 3518 postal code areas in Germany.** Retail sales data were provided by SymphonyIRI Group GmbH, Germany.
(CSV)

Dataset S2 **The first column in this data file indicates anonymous food product IDs (the number before the underscore in the naming convention) and the index number for experimental runs (the number after the underscore) up to 50 replications.** In each simulation for a “suspect” product, we sampled 100 postal code areas shown in column B to CW used to represent the locale of case reports identified for a synthetic outbreak. Retail sales data were provided by SymphonyIRI Group GmbH, Germany.
(RAR)

Figure S1 a–b **Success rate and likelihood ratio for individual products (as contaminated product).**
(TIF)

Figure S2 a–c
**Success rate as a function of maximum correlation (Spearman's** 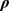 **) for (a) 10 case reports, (b) 20 case reports, and (c) 50 case reports.** For large correlations, the contaminated product cannot always be uniquely determined.(TIF)

Text S1 **Derivation of likelihood.**
(DOC)
