# Correction: A Likelihood-Based Approach to Identifying Contaminated Food Products Using Sales Data: Performance and Challenges

**DOI:** 10.1371/journal.pcbi.1003999

**Published:** 2014-11-03

**Authors:** The PLOS Computational Biology Staff

The author contributions of the manuscript are incorrect, the correct contributions are:

Conceived and designed the experiments: SE KH JK JL.

Performed the experiments: KH MF CT SE AH.

Analyzed the data: BA CT KH AH AK JL MF.

Contributed materials and analysis tools: SE AH KH JK CT JL.

Wrote the paper: SE KH JD MF JK JL.

Preprocessed/organized raw food sales data: CT MF BA AK.

Designed/wrote likelihood algorithm: SE JL.

Designed and performed clustering analysis: AH CT.

Proposed this use of retail data with public health data: MF JK.
