# Supplementary figures and images for: A Likelihood-Based Approach to Identifying Contaminated Food Products Using Sales Data: Performance and Challenges
Source: PLoS Comput Biol. 2014 Jul 3;10(7):e1003692. doi: 10.1371/journal.pcbi.1003692 (PMC4080998; doi:10.1371/journal.pcbi.1003692)

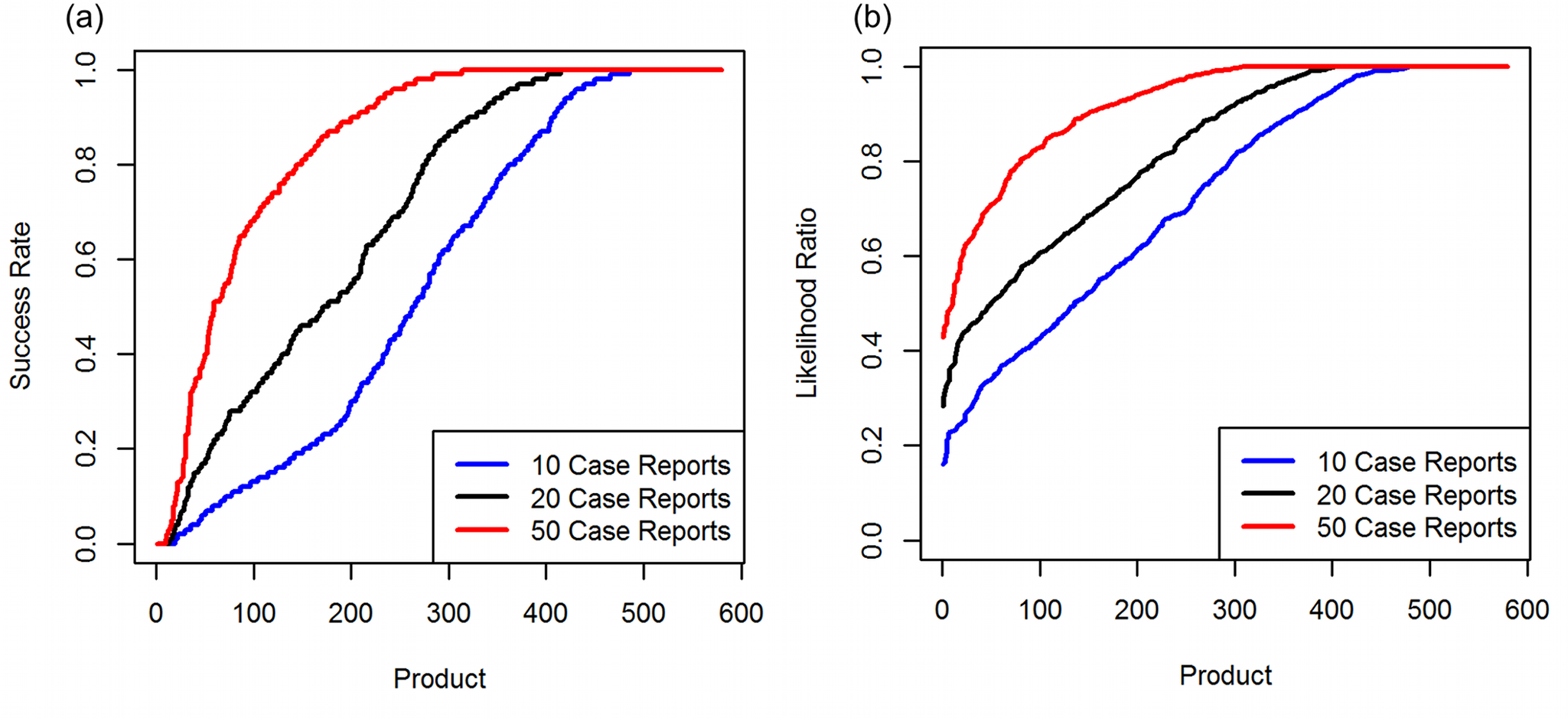

Supplement: Figure S1 a–b — Success rate and likelihood ratio for individual products (as contaminated product). (TIF) [file pcbi.1003692.s003.tif]

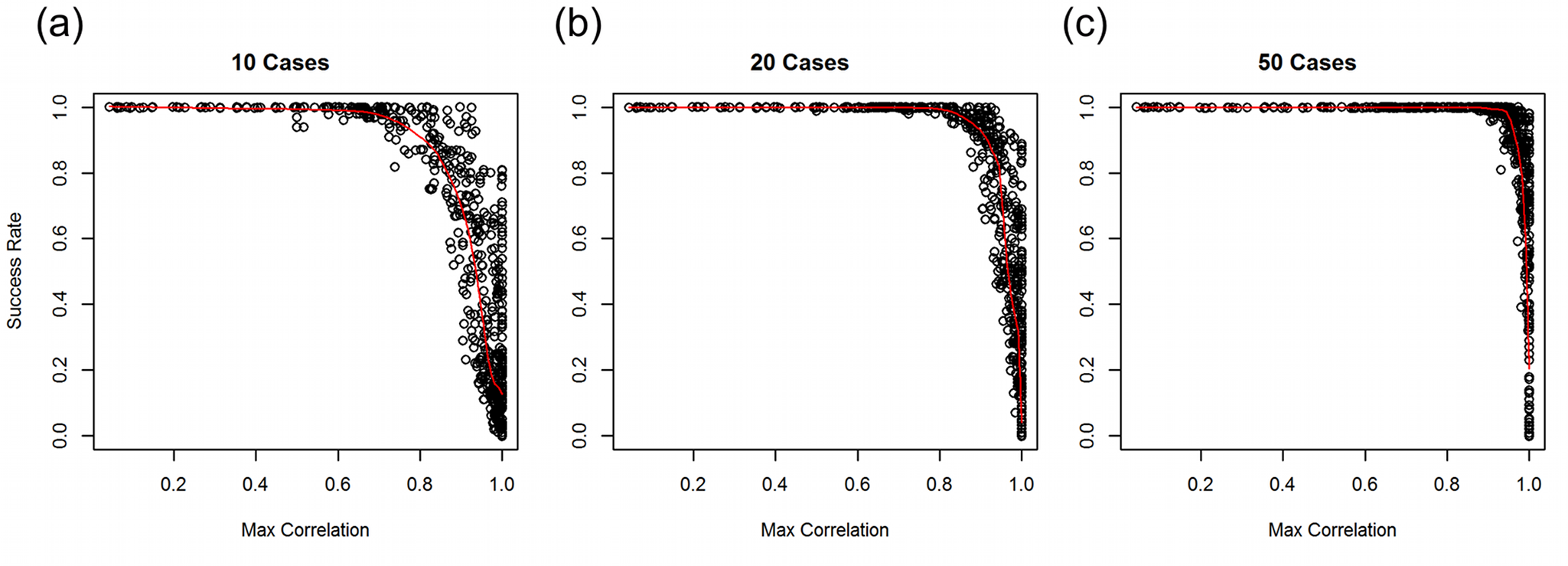

Supplement: Figure S2 a–c — Success rate as a function of maximum correlation (Spearman's ) for (a) 10 case reports, (b) 20 case reports, and (c) 50 case reports. For large correlations, the contaminated product cannot always be uniquely determined. (TIF) [file pcbi.1003692.s004.tif]
