# Supplementary material for: A Likelihood-Based Approach to Identifying Contaminated Food Products Using Sales Data: Performance and Challenges
Source: PLoS Comput Biol. 2014 Jul 3;10(7):e1003692. doi: 10.1371/journal.pcbi.1003692 (PMC4080998; doi:10.1371/journal.pcbi.1003692)
Supplement: Text S1 — Derivation of likelihood. (DOC) [file pcbi.1003692.s005.doc]

Text S1. Derivation of Likelihood

Let $\theta=\left\langle\text{0,...,0,1,0,...,}\left.0\right\rangle\right.$ be a vector in which the$j^{th}$component is one if distributed food $j$is contaminated:

$$=P(i\text{ is infected and }i\text{ lives in postal code }r\text{ | }\theta)$$

$$=P(i\text{ lives in postal code }r)\cdot P(i\text{ is infected | }i\text{ lives in postal code }r\text{, }\theta)$$

where $\phi_{r}$ denotes the population density in postal code $r$. An individual can be a determined carrier of infection with certainty $\xi$. Each distributed food product $j$is associated with a retail store.

Then having observed a set of reported cases$D$, the likelihood becomes:

Assuming $f\left(i,k\right)$denotes the probability that customer$i$ shops at store $k$ given lives at $x\text{, }y$, we arrive at the following objective function:

Since the first two terms are constant, we can simplify to:

$$O(\theta|D)=\sum_{i\in D} \sum_{j} \theta_{j}\log\left[\sum_{k\in R_{j}} f\left(i,k\right)\right]$$

We maximize this function to determine the most likely contaminated food.

The performance of the likelihood measure for individual products can vary as shown in supplemental Figure S1. The figure shows three different examples of success rate as a function of number of case reports for three different products.

From supplemental Figure A2, Success Rate as a function of Spearman’s $\rho$, it is evident that for large correlations (>0.8), the contaminated product cannot always be uniquely determined.

This figure also demonstrates that as the maximum pairwise correlation between a contaminated product and another product increases, the number of cases required to *reduce* the suspect product set size to a manageable number (e.g., below 10) increases. This is associated with a corresponding decrease in success rate at high correlation. In addition, if the contaminated food and the product most related to it have correlation less than 0.6, then the likelihood method can uniquely identify this contaminated product with 95% confidence. Unique identification (“success”) is equivalent to a suspect product set size of 1.
